# Poor Reproducibility of Allergic Rhinitis SNP Associations

**DOI:** 10.1371/journal.pone.0053975

**Published:** 2013-01-30

**Authors:** Daniel Nilsson, Anand Kumar Andiappan, Christer Halldén, Chew Fook Tim, Torbjörn Säll, De Yun Wang, Lars-Olaf Cardell

**Affiliations:** 1 Division of ENT Diseases, Department of Clinical Science, Intervention and Technology, Karolinska Institutet, Stockholm, Sweden; 2 Biomedicine, Kristianstad University, Kristianstad, Sweden; 3 Department of Biological Sciences, National University of Singapore, Singapore, Singapore; 4 Singapore Immunology Network (SIgN), Singapore, Singapore; 5 Department of Cell and Organism Biology, Lund University, Lund, Sweden; 6 Department of Otolaryngology, National University of Singapore, Singapore, Singapore; Emory University School of Medicine, United States of America

## Abstract

Replication of reported associations is crucial to the investigation of complex disease. More than 100 SNPs have previously been reported as associated with allergic rhinitis (AR), but few of these have been replicated successfully. To investigate the general reproducibility of reported AR-associations in candidate gene studies, one Swedish (352 AR-cases, 709 controls) and one Singapore Chinese population (948 AR-cases, 580 controls) were analyzed using 49 AR-associated SNPs. The overall pattern of *P-*values indicated that very few of the investigated SNPs were associated with AR. Given published odds ratios (ORs) most SNPs showed high power to detect an association, but no correlations were found between the ORs of the two study populations or with published ORs. None of the association signals were in common to the two genome-wide association studies published in AR, indicating that the associations represent false positives or have much lower effect-sizes than reported.

## Introduction

Allergic rhinitis (AR) is the most common allergic disease affecting more than 500 million people worldwide and is characterized by nasal symptoms including sneezing, rhinorrhea and nasal blockage. AR is also associated with an IgE-mediated immune response against allergens [1]. The development of AR is due to a complex interaction between both environmental and genetic factors. The heritability for AR has been estimated to be as high as 70–90% [2], [3].

In AR a number of case-control and family-based association studies as well as linkage studies have identified a number of SNPs and chromosomal regions potentially associated with the disease. Until 2010 an absolute majority of the genetic studies of AR were candidate gene studies. The few exceptions were cases of classical linkage analysis. In 2011 a genome-wide association study (GWAS) [4] and a GWAS meta-study [5] were published in addition to a number of candidate gene studies. Thus, the current list of implicated SNPs is clearly dominated by results from early candidate gene studies. In total, this has resulted in more than 100 single nucleotide polymorphisms (SNPs) reported as associated with AR. However, the investigations that have resulted in this list of SNPs are very heterogeneous with respect to sample sizes, type of study populations and phenotype definitions.

Case-control association tests have become a very popular method to search for genetic variants in linkage disequilibrium with risk factors (http://www.wtccc.org.uk). Today, a large number of traits/diseases have been investigated using this technique e.g. diabetes, schizophrenia, coronary diseases and various forms of cancer. Over the past 15 years standards have shifted towards larger sample sizes of both cases and controls. It has also become apparent that an association can only be considered reliable if it has been properly replicated. In a study based on self-reported data on 50 medical phenotypes from a cohort of more than 20 000 genotyped individuals, approximately 75% of expected associations were successfully replicated [6]. One observation was that different traits and diseases varied drastically with respect to their reproducibility of reported associations, probably reflecting inflation of the effects in some reports and differences among diseases in the likelihood of proper disease classification. In a systematic investigation aiming for replication of reported risk-associated polymorphisms in prostate cancer, 18 out of 50 SNPs were successively replicated [7], whereas only 10 out of 93 and 5 out of 44 SNPs were replicated in two different replication studies in asthma [8], [9]. Thus, in the ongoing investigation of a disease it is of importance to monitor the general reproducibility of reported associations. In AR no such evaluation has previously been performed.

In this study we have investigated the general reproducibility of reported SNP associations from previous candidate gene studies using two different study populations, one Swedish and one Singapore Chinese. The result shows poor reproducibility of AR SNP associations.

## Results and Discussion

To investigate the reproducibility of reported associations from previous candidate gene studies in AR, two study populations, one from Sweden (352 AR-cases, 709 controls) and one from Singapore (948 AR-cases, 580 controls), were analyzed using a set of 49 SNPs reported as associated with AR. These SNPs constitute a subset of a total of 116 AR-associated SNP markers compiled from 52 different studies (Table S1 and S2). The present study investigates the same phenotype (AR) and the same SNP markers that were reported in the initial studies.

Association test results are summarized in Table 1, Table S3, S4, S5 and S6. In the Singapore Chinese population (CP), 45 SNPs were analyzed. The distribution of *P*-values showed a very good fit to the expected distribution in the absence of an effect. The lowest *P*-value was 0.003 for rs3761547 in the *FOXP3* gene and no SNP showed a false discovery rate (*Q-*value) below 0.1. The 49 SNPs analyzed in the Swedish population (SP) also showed a good fit to the expected distribution of *P*-values in the absence of an effect. There was, however, a slight overrepresentation of *P*-values below 0.01. The lowest *P*-value (*P = *0.00084, *Q = *0.033) was found for rs4732416 in *CCL24* when testing for genotypic effects. However, in the corresponding test of allelic effects the *P*-value was 0.75 and two additional SNPs in this gene showed no significant associations. In the *FOXJ1*-gene the rs880213 SNP showed *P*-values below 0.01 both at the allele and genotype levels. The corresponding *Q*-values were 0.28 and 0.085. Significant *P*-values were observed in both populations for rs20541 in *IL13* and for rs13340504 in *CCL24*. In both cases, however, significant *P*-values were observed for the association test in CP and the skin prick test (SPT) in SP. Thus, the overall pattern of *P-* and *Q-*values in the present study indicates that very few if any of the SNPs are associated with AR.

**Table 1:** Observed and expected numbers of *P*-values for the different association tests in the Swedish population (n = 1061) and the Singapore Chinese population (n = 1528).

*P*-value range		Swedish population					Singapore Chinese population				
		Allele	Genotype	Birch	Timothy	Total	Allele	Genotype	*B. tropicalis*	*D. pteronyssinus*	Total
1.0–0.1	Obs/Exp	45/44	45/44	42/43	42/43	174/174	41/41	39/41	43/41	41/41	166/162
	*Q*-values	0.88–0.99	0.59–0.74	0.73–0.97	0.50–0.80	0.50–0.99	0.88–0.97	0.53–0.77	0.76–0.90	0.42–0.97	0.42–0.97
0.1–0.05	Obs/Exp	3/2.5	2/2.5	4/2.4	4/2.4	13/9.8	2/2.3	4/2.3	1/2.3	1/2.3	7/9.2
	*Q*-values	0.88–0.93	0.59	0.73	0.45–0.49	0.45–0.93	0.88	0.46	0.76	0.42	0.42–0.88
0.05–0.01	Obs/Exp	0/2.0	0/2.0	1/1.9	1/1.9	2/7.8	2/1.8	2/1.8	0/1.8	2/1.8	6/7.2
	*Q*-values	−	−	0.73	0.45	0.45–0.73	0.60	0.46	−	0.15–0.19	0.15–0.60
0.01–0.001	Obs/Exp	1/0.44	1/0.44	1/0.43	1/0.43	4/1.7	0/0.40	0/0.40	2/0.40	1/0.40	3/1.6
	*Q*-values	0.28	0.085	0.23	0.12	0.085–0.28	−	−	0.11–0.13	0.14	0.11–0.14
0.001	Obs/Exp	0/0.05	1/0.050	0/0.050	0/0.050	1/0.20	0/0.040	0/0.040	0/0.040	0/0.040	0/0.16
	*Q*-values	−	0.033	−	−	−	−	−	−	−	−

Obs/Exp = Observed number of *P*-values/Expected number of *P*-values in the absence of effect.

*Q*-values = Observed *Q*-values calculated according to Storey JD (2002) A direct approach to false discovery rates. J R Stat Soc Series B Stat Methodol 64∶479–498.

The lack of replication can have several explanations: the previously reported effects may simply not be present in the populations investigated here, the original reports may be false positive associations (type I error), or the reported odds ratios (ORs) may be inflated due to chance (the “winners curse” effect). Also, the use of different clinical definitions compared to the original reports resulting in enrichment of cases with different clinical subtypes makes replication difficult. In addition, interactions with other factors such as age, sex and urban/rural residency may have an impact on replication. To investigate the ability of the present study to detect the previously reported associations, we estimated the power for the allele-based test in the present investigation. Given calculated or published ORs and the sample sizes used in the present study, the power was calculated at significance levels 0.05 and 0.001. The results are shown in Table 2 where most SNPs show high power to detect an association. The expected number of significant *P*-values is calculated as the sum of the power estimates with standard deviation = (∑*power*_i_(1-*power*_i_))^1/2^. The expected number varies between 16.3 and 22.3 with standard deviations between 1.0 and 1.8. Since the observed number of significant *P*-values varies between 0 and 2 (Table 1), the expected numbers exceed the observed by many standard deviations, i.e. if the published ORs would have been true a number of significant results would have been expected. However, it is not an uncommon observation that early association studies report highly inflated ORs. Although the observed results are not surprising, the present investigation is the first systematic study of this aspect in AR.

**Table 2:** Power estimates in present investigation given odds ratios extracted or calculated from published data.

Gene	SNP ID	Polymorphism	Population	Population size (case; control)	OR†	Power (SP)‡		Power (CP)‡		Reference*
						0.05	0.001	0.05	0.001	
*IL28RA*	rs7525481	g.32349 G>A	Korean	267; 559	0.74	0.72	0.20	0.98	0.76	[10]
*FCGR2A*	rs1801274	R131R	Turkish	180; 234	1.93	1.00	1.00	1.00	1.00	[11]
*CD55 (DAF)*	rs10746463		Japanese	684; 346	0.73	0.86	0.40	−	−	[12]
*CCR3*	rs4987053	51T/C	Japanese	151; 157	7.75	1.00	1.00	1.00	1.00	[13]
*IL2*	rs2069762	−330T/G	German	318; 322	1.29	0.72	0.22	0.91	0.49	[14]
*IL13*	rs1800925	C-1112T	Spanish	146; 50	0.16	1.00	1.00	1.00	1.00	[15]
*IL13*	rs20541	R130Q	Spanish	146; 50	1.52	0.97	0.73	1.00	0.98	[15]
*HAVCR2*	rs1036199	4259G>T	Korean	201; 319	1.54	0.98	0.75	0.16	0.00	[16]
*CCL26*	rs2302009	2497T>G	Korean	178; 281	2.87	1.00	1.00	1.00	1.00	[17]
*CCL24*	rs2302004	179T>C	Korean	178; 281	1.10	0.19	0.01	0.22	0.02	[17]
*IL33*	rs1929992		Japanese	170; 100	1.41	0.95	0.63	1.00	0.90	[18]
*GATA3*	rs1269486		Han Chinese	109; 112	0.35	1.00	1.00	1.00	0.97	[19]
*MS4A2*	rs569108	Glu237Gly	Japanese	233; 100	1.47	0.17	0.01	0.99	0.81	[20]
*IL18*	rs187238	−137G>C	Swiss	1105; 2953	1.12	0.20	0.01	0.17	0.01	[21]
*IL18*	rs187238	−137C	German	25; 80	2.72*	1.00	1.00	1.00	1.00	[22]
*RNASE3*	rs2233860		Korean	440; 478	1.36	0.78	0.30	0.87	0.41	[23]
*IL4R*	rs1805010	Ile50Val	Japanese	145; 206	0.62	1.00	0.96	1.00	1.00	[24]
*IL4R*	rs1805011	Glu375Ala	Japanese	145; 206	0.35	1.00	1.00	1.00	1.00	[24]
*NOD2*	rs5743266		German	488; 978	0.82*	0.43	0.06	0.08	0.00	[25]
*NOD2*	rs2066842		German	488; 978	0.80*	0.49	0.08	0.08	0.00	[25]
*NOD2*	rs2066844	C2104T	German	154; 1765	1.73*	0.53	0.13	−	−	[26]
*NOD2*	rs2066845	G2722C	German	154; 1765	3.16*	0.87	0.46	−	−	[26]
*CCL5*	rs2280788	−28C/G	Korean	151; 278	1.58	0.43	0.08	0.98	0.78	[27]
*CCL5*	rs2107538	−403G/A	Korean	151; 278	1.42	0.85	0.41	1.00	0.89	[27]
*EPX*	rs2240815	3979A/G	Czech	294; 319	1.28	0.76	0.27	0.85	0.39	[28]
*FOXJ1*	rs3192453	g.3375G>C	Korean	295; 418	0.62	0.78	0.25	0.98	0.78	[29]
*FOXJ1*	rs880213	g. −460C>T	Korean	295; 418	0.64	0.89	0.43	0.95	0.64	[29]
*ADAM33*	rs2787094		Chinese Han	128; 151	4.01	1.00	1.00	1.00	1.00	[30]
*ADAM33*	rs2280089	12540C/T	Japanese	95; 95	0.32	1.00	1.00	1.00	1.00	[31]
*ADAM33*	rs2280089	12540C/T	Chinese Han	128; 151	1.91	1.00	0.98	1.00	0.99	[30]
*ADAM33*	rs2280090	12462C/T	Japanese	95; 95	0.28	1.00	1.00	1.00	1.00	[31]
*ADAM33*	rs2280090	12462C/T	Chinese Han	128; 151	0.40	1.00	0.99	1.00	1.00	[30]
*ADAM33*	rs511898	7575G/A	Japanese	95; 95	0.60	1.00	0.96	1.00	1.00	[31]

† Odds ratios (OR) as reported in reference (*) or calculated for minor allele given published data.

‡ Power calculations at different levels of significance in the Swedish (SP) and the Singapore Chinese (CP) population. The significance level 0.001 corresponds approximately to the Bonferroni correction level.

If the originally reported significant ORs are due to a “winners curse” effect, the power in our analysis may be insufficient. If this is true, an effect could perhaps still be traced via a correlation of effects between investigations. Spearman correlation coefficients were therefore calculated from the ORs for allelic effects for the different sets of data. Comparing data from the SP with published data from the other European populations, a non-significant result was obtained with a correlation coefficient of 0.08 (*P = *0.84). In the corresponding comparison between data obtained from the CP and published data from other Asian populations the correlation coefficient was higher but still non-significant (R = 0.33 and *P = *0.19). Finally, the concordance between SP and CP was calculated. Also this comparison gave a low and non-significant correlation coefficient (R = 0.03 and *P* = 0.87).

A third strategy to investigate potential associations would be to test for identical trends among SNPs. This was done by calculating the number of concordant SNPs, i.e. SNPs for which both ORs were either larger than 1 or less than 1, as well as the number of discordant SNPs, i.e. SNPs where one OR was less than 1 and the other was larger than 1. In the comparison between the Swedish and Singapore results, 23 SNPs were concordant and 20 were discordant, which of course is not significantly different from equal proportions. Thus, even at this basic level no sign of effects of the investigated SNPs could be detected.

Several GWAS have been performed to identify genetic variants for asthma and related phenotypes whereas only one study has been performed aiming specifically for AR [4]. In the latter no associations were detected at a genome-wide significance level, and only two at a suggestive significance level. A genome-wide meta-analysis based on self-reported phenotyping identified few genetic variants associated with AR in spite of analyzing 2.2 million SNPs in close to 4000 AR cases and 9000 controls [5]. Only one locus reached genome-wide significance and six suggestive loci were identified. When this data set was used in a candidate gene approach analyzing 164 genes only one locus was identified as suggestive for the AR phenotype [5]. Comparing these two GWAS there were no significant association signals in common, and the ORs identified for the significant SNPs were in the size ranges 0.6–1.4 and 1.1–1.3, respectively, in the two studies [4], [5]. This is in contrast to the previously reported associations listed in Table S1 and S2 where many show much stronger effects (OR = 0.16–7.8). In addition, none of these associations are detected in the GWAS described above.

In conclusion, the overall pattern of *P-* and *Q-*values indicates that very few, if any, of the 49 investigated SNPs are significantly associated with AR in our populations. If associations do exist they must have much lower effect sizes than reported. This is in agreement with the results of the GWAS where reported effect sizes are fairly modest. The results above do not exclude that certain cases may represent true associations. Due to the necessary compensation for the multiple testing involved, the larger the number of SNPs that are included in a study, the harder it is to detect true associations. The obvious candidates are of course the SNPs with low *P*-values mentioned above. In these cases, the likelihood of an association is strengthened if the ORs in CP, SP and the original publication are concordant. This is not the case for the SNPs in *CCL24*, *FOXJ1* and *IL33.* The strongest case using this criterion would be rs20541 in *IL13* where there is complete concordance in the direction of the ORs between both populations and the original result. Thus, even though this SNP did not yield the lowest *P*-value, it is potentially the best candidate for future investigations. There are a number of other reasons for the lack of replication, such as the use of different clinical definitions or that different studies may be enriched in cases with different clinical subtypes. This indicates that future GWAS or candidate gene studies favorably would use much larger population sizes and more careful phenotyping using strictly defined guidelines than what has been used in the past. In addition, alternative strategies for the identification of AR-associated genes, such as exome sequencing, may be more successful strategies.

## Materials and Methods

### Ethics Statement

This study was approved by the Ethics Committee of the Medical Faculty, Lund University and the Institutional Review Board of National University of Singapore and written informed consent was obtained from all subjects. This study is also in compliance with the Helsinki declaration.

### Study Populations and Phenotype Definitions

The Swedish study population was recruited at Malmö University hospital (Malmö, Sweden) between the years 2003 and 2009 and consists of unrelated subjects from the general population. It is comprised of 360 AR-cases (169 females, 191 males, mean age 33 years) and 720 controls (294 females, 426 males, mean age 43 years) with no atopy and allergic symptomology. All cases were patients at the allergy clinic and were diagnosed with symptomatic birch and/or timothy grass pollen induced intermittent AR. Both cases and controls were of Caucasian origin, with both parents born in Sweden. In the Swedish population skin prick tests (SPT) [32] were performed with a standard panel of 11 common airborne allergens (ALK-Abelló, Hørsholm, Denmark). This study population has previously been analyzed in several AR studies [33], [34], [35].

The Singapore Chinese population was collected in Singapore over multiple volunteer recruitment drives and consists of unrelated subjects. In the Singapore Chinese population SPT was using a panel consisting of common allergens in Singapore such as *Dermatophagoides pteronyssinus* and *Blomia tropicalis*. The population used in this study consists of 1024 AR cases (549 females, 475 males, mean age 22 years) with symptomatic dust mite induced AR and 605 controls (449 females, 156 males, mean age 22 years) with no atopy and allergic symptomology. This study population has previously been analyzed in several AR studies [4], [35], [36], [37], [38].

Diagnostic procedures for the study populations included personal interview of medical history and SPT or Phadiatop tests and were performed using standard panels of common airborne allergens. SPT were performed on the volar side of the forearm with saline buffer as negative and histamine chloride (10 mg/ml) as positive controls. A wheal reaction diameter of ≥3 mm was considered a positive SPT response. SPT was only performed if the AR cases had not taken any anti-allergic drugs for at least 3 days prior to the test. Atopy is defined as a positive SPT reaction to either one of allergens tested. AR is thus diagnosed based on the presence of atopic status and typical AR symptoms as defined by the Allergic Rhinitis Impact on Asthma (ARIA) guidelines i.e., two or more AR symptoms (nasal congestion, rhinorrhea, nasal itching, sneezing) persisting for four or more days per week during the past year [1], [39]. Conversely, the non-allergic controls are defined by having no atopy and no typical AR symptoms.

### Literature Mining and SNP Selection

PubMed (www.ncbi.nlm.nih.gov/pubmed/) was used to search for reports on AR associations using the search terms: (rhinitis OR hay fever) AND (association OR “case control”) AND (polymorphism OR SNP OR mutation) AND English [language]. Studies that only reported skin prick test scores, associations with severity or related phenotypes such as IgE levels, but not allergic rhinitis *per se* were not considered in this study. The first literature search was conducted 2010-03-16 and served as a base for selection of 49 randomly chosen SNPs previously reported to be associated with AR (*P*<0.05). A second literature search was made 2012-06-26, using the same search terms as described above, to make an up-to-date list containing all previously associated SNPs with AR (Table S1 and S2).

### Genotyping of Candidate Polymorphisms

Genomic DNA was extracted from blood collected in EDTA using QIAamp DNA Blood Maxi or Mini kits (Qiagen, Hilden, Germany) and DNA concentrations determined by fluorometry using PicoGreen (Molecular Probes, Eugene, OR, USA). Genotypes were determined using the Sequenom MassARRAY MALDI-TOF system. The system analyzes allele-specific primer extension products using mass-spectrometry. Assay design was made using the MassARRAY Assay Design ver. 2.0 software (Sequenom Inc, San Diego, CA, USA). Primers were obtained from Metabion GmbH, Germany and all reactions were run under the same conditions except for the primer annealing temperature of the primary PCR. PCR reactions were performed in a total volume of 6 µl containing 2.5 ng of template DNA, 1.25X HotStar Taq PCR buffer, 0.15 units of HotStar Taq polymerase, 3.5 mM MgCl_2_, 0.5 mM dNTPs and 100 nM of each primer. Amplifications were performed using GeneAmp 9700 machines with dual-384 heads as follows: 95°C for 15 min, 45 cycles of 95°C for 20 s, 56°C, 60°C or 64°C for 30 s, 72°C for 60 s and finally 72°C for 3 min. Dephosphorylation of unincorporated dNTPs was achieved using shrimp alkaline phosphatase. Concentrations of individual hME primer pairs were adjusted to even out peak heights in the mass spectrum. The extension reactions were then made by mixing the adjusted MassEXTEND primer mix (containing approximately 1 µM of each primer) with hME EXTEND mix containing buffer and 50 µM of each d/ddNTP mix and 1.25 units of Thermo Sequenase. PCR amplification of hME reactions was performed as follows: 94°C for 2 min and 99 cycles of 94°C for 5 s, 52°C for 5 s and 72°C for 5 s. The samples were then manually desalted by using 6 mg of Clean Resin and a dimple plate and subsequently transferred to a 384-well SpectroCHIP using a nanodispenser.

Analyzing 49 SNPs, the total genotyping rate in the Swedish population was 99.4% in 352 (168 female, 184 male) cases and 709 (291 female, 418 male) controls after removal of low-quality DNA samples. In the Chinese population, the total genotyping rate after removal of low-quality data was 99.7% in 948 (514 female, 434 male) cases and 580 (440 female, 140 male) controls. Only 45 SNPs were polymorphic in the Singapore Chinese population.

### Statistical Analysis

Statistical analyses were made using R statistical software [40] and the genetics package [41]. Genotype frequencies were calculated and tested for Hardy-Weinberg equilibrium in both cases and controls. Allele and genotype frequencies were then investigated for association with AR using a *χ* ^2^-homogeneity test. ORs and 95% confidence intervals were estimated by using the most common allele as the referent and are reported for each minor allele. The Kruskal-Wallis rank sum test was used for analysis of associations between genotypes and SPT-scores. The score is defined as the size of the wheel reaction in relation to histamine. In order to compensate for the effect of multiple testing, *Q*-values was calculated using the software QVALUE (ver.1.0) [42]. One of the assumptions underlying the calculation of the *Q*-values is that the *P*-values will be uniformly distributed under the null hypothesis. We have calculated the number of expected *P*-values in the different ranges, with a higher resolution for the lower values.

Power calculations were made through simulations for the association tests of allele effects. The calculations were made separately for the Swedish and Singapore populations. For each SNP we simulated a data set with the actual numbers of cases and controls of the respective population, using the published or calculated ORs (given published data) and the allele frequencies observed in our populations. A total of 10 000 runs were made in each case. For these, a full data set was simulated and the *χ* ^2^-value was calculated. The number of times the test quantity exceeded the critical values for 0.05 and 0.001 was subsequently scored.

## Supporting Information

Table S1 **Genes and SNPs reported to be associated with allergic rhinitis in case-control studies.**
(XLS)

Table S2 **Genes and SNPs reported to be associated with allergic rhinitis in family-based studies.**
(XLS)

Table S3 **Minor allele frequencies (MAF) and P-values for Hardy-Weinberg (HW), association and skin prick test in the Singapore Chinese population.**
(XLS)

Table S4 **Observed odds ratios (OR) and 95% confidence intervalls (CI) in the Singapore Chinese population.**
(XLS)

Table S5 **Minor allele frequencies (MAF) and P-values for Hardy-Weinberg (HW), association and skin prick test in the Swedish population.**
(XLS)

Table S6 **Observed odds ratios (OR) and 95% confidence intervalls (CI) in the Swedish population.**
(XLS)
